# The Relationship Between Cardiometabolic Index and New-Onset Diabetes in Adults Aged Over 45: A Longitudinal Analysis Based on CHARLS

**DOI:** 10.1155/ije/2259853

**Published:** 2025-07-29

**Authors:** Xiaoqin Chen, Zhitong Li, Tao Liu, Xingyu Li, Xuhui Li, Ruixue Duan, Shiwei Liu

**Affiliations:** ^1^Third Hospital of Shanxi Medical University, Shanxi Bethune Hospital, Tongji Shanxi Hospital, Shanxi Academy of Medical Sciences, Taiyuan, Shanxi, China; ^2^Department of Endocrinology, Third Hospital of Shanxi Medical University, Shanxi Bethune Hospital, Tongji Shanxi Hospital, Shanxi Academy of Medical Sciences, Taiyuan, Shanxi, China; ^3^Department of General Surgery, Third Hospital of Shanxi Medical University, Shanxi Bethune Hospital, Tongji Shanxi Hospital, Shanxi Academy of Medical Sciences, Taiyuan, Shanxi, China; ^4^School of Forensic Medicine, Shanxi Medical University, Jinzhong, Shanxi, China

**Keywords:** cardiometabolic index, China health and retirement longitudinal study, diabetes mellitus, risk prediction

## Abstract

**Objective:** The cardiometabolic index (CMI) serves as a comprehensive indicator of metabolic risk. Recent findings indicate a potential association between CMI and the likelihood of diabetes onset. The objective of this research is to explore the correlation between initial CMI values and the occurrence of newly diagnosed diabetes mellitus in individuals aged 45 years and above in China.

**Methods:** In this research, data were sourced from the China Health and Retirement Longitudinal Study (CHARLS). To evaluate the association between baseline CMI and the incidence of newly diagnosed diabetes, multivariate logistic regression models were employed, with adjustments made for various potential confounding factors. Additionally, stratified analyses evaluated subgroup variations, and mediation analysis explored pathways through which CMI influences diabetes risk.

**Results:** A cohort of 4,944 participants was followed, with 786 diagnosed with new-onset diabetes. The occurrence of diabetes escalated with increasing CMI quartiles, with Q4 showing a 141% increased risk (OR 2.41, 95% CI, 1.94–3.02) in unadjusted models. This association remained significant after adjustment. Stratified analyses found that dyslipidemia modified the CMI–diabetes association. CMI, age, BMI, HbA1c, and hypertension were identified as independent predictors of new-onset diabetes. Mediation analysis revealed that HbA1c mediated 9.70% of the CMI–diabetes relationship.

**Conclusion:** In summary, our research establishes a connection between increased levels of CMI and the onset of diabetes, suggesting its potential as a predictive tool. The findings highlight CMI's role in assessing diabetes risk and call for targeted interventions. Future research should validate these associations and explore underlying mechanisms for better prevention strategies.

## 1. Introduction

Diabetes mellitus poses a substantial public health issue worldwide, with its incidence rising at a concerning pace, especially among individuals in middle and older age groups [1, 2]. Type 2 diabetes mellitus, recognized as the prevalent variant of diabetes, is a complex metabolic condition predominantly marked by insulin resistance (IR) and persistent hyperglycemia [3]. The burden of diabetes extends beyond individual health, contributing to substantial healthcare costs, heightened illness rates, and diminished life quality. Considering the progressive characteristics of the disease, it is essential to promptly identify individuals who are at risk to facilitate timely interventions, preventive measures, and effective management strategies. However, traditional risk assessment methods often focus on isolated factors such as obesity, dyslipidemia, and hypertension, rather than evaluating their cumulative impact on metabolic health [4]. This limitation underscores the need for composite indices that integrate multiple risk factors into a single, predictive measure.

The cardiometabolic index (CMI)—calculated as waist-to-height ratio multiplied by triglyceride (TG)/HDL ratio—integrates visceral adiposity and dyslipidemia into a unified metric for comprehensive cardiometabolic risk assessment [5]. Unlike traditional single-factor indicators or specialized indices (e.g., triglyceride–glucose [TyG] index for glucose–lipid interplay or visceral adiposity index [VAI] for body mass index [BMI]–based adiposity), CMI captures synergistic interactions between obesity-driven lipid dysregulation and IR [6, 7]. This makes it particularly valuable for diabetes risk stratification in middle-aged/elderly populations, where visceral fat accumulation is a dominant pathophysiological factor. CMI predicts diverse outcomes including all-cause mortality, diabetes progression [8], hypertension, hyperuricemia, and cardiovascular events [9–11], positioning it as a significant predictor of metabolic and cardiovascular risk. Therefore, CMI assessment in middle-aged and older adults offers a promising approach for early intervention and risk stratification in metabolic disorders.

The aging process is intrinsically linked to metabolic deterioration, with the middle-aged and elderly population experiencing an increased susceptibility to IR and glucose dysregulation [12]. This susceptibility is intensified by lifestyle choices, including a lack of physical activity, unhealthy eating patterns, and elevated levels of visceral fat [13]. Given that early metabolic changes often precede the clinical onset of diabetes by several years, identifying reliable predictors of disease progression in this demographic is imperative for effective prevention strategies. Longitudinal studies are essential in this regard, as they allow for the examination of temporal relationships between metabolic indicators and disease incidence. However, the majority of current research focused on diabetes forecasting is based on cross-sectional data, which restricts the capacity to determine causal relationships and monitor metabolic alterations over a period [14]. The China Health and Retirement Longitudinal Study (CHARLS) offers a valuable opportunity to explore these associations within a representative sample of middle-aged and older individuals in China. This nationwide survey gathers comprehensive longitudinal information regarding health conditions, socioeconomic factors, and lifestyle choices, rendering it an optimal resource for assessing the predictive influence of CMI on the onset of diabetes [15]. Considering the swift increase in the aging demographic in China, alongside the growing incidence of diabetes, it is essential to comprehend the correlation between CMI and the emergence of new diabetes cases. This understanding holds significant relevance for the formulation of public health strategies and the enhancement of clinical practices.

Despite the increasing acknowledgment of CMI as a potential metabolic risk marker, research examining its long-term predictive capacity for diabetes is still limited. Most investigations have primarily concentrated on specific metabolic components or short-term associations, rather than evaluating the cumulative effects of CMI over extended periods. Furthermore, despite the encouraging results from previous research, the majority of these studies have primarily focused on populations in Western countries, especially the United States and Japan. This has resulted in a notable deficiency in the literature concerning the role of CMI in forecasting the onset of diabetes among middle-aged and older adults [16, 17]. While recent studies have explored CMI–diabetes associations in Chinese cohorts [16, 18], our study extends this evidence by: (1) utilizing longitudinal CHARLS data with biochemical validation of diabetes; (2) evaluating mediation pathways (e.g., inflammation, hypertension); and (3) identifying dyslipidemia as a key effect modifier—a finding previously unreported. This gap is particularly pronounced in Chinese populations, where relevant studies are notably scarce. The applicability of CMI across diverse populations and age groups necessitates further validation. Considering the intricate nature of metabolic syndrome alongside the underlying mechanisms contributing to the development of diabetes, a more comprehensive understanding of how CMI contributes to disease progression could improve risk stratification models and guide targeted intervention strategies.

The objective of this research is to examine the association between CMI and the onset of diabetes in adult individuals aged 45 years and above, utilizing longitudinal data obtained from CHARLS. Specifically, we seek to: (1) investigate the relationship between initial CMI levels and the occurrence of diabetes over a specified duration and (2) determine whether CMI serves as an independent predictor of diabetes onset following the adjustment for possible confounding variables, including age, lifestyle factors, and pre-existing health conditions. By addressing these objectives, the objective of this investigation is to offer new perspectives on the significance of CMI in predicting diabetes, as well as to aid in the creation of improved screening methodologies for the timely identification of the condition. The results may carry significant consequences for individualized risk evaluation, clinical judgment, and public health strategies designed to alleviate the increasing prevalence of diabetes in aging demographics.

## 2. Materials and Methods

### 2.1. Study Population

CHARLS represents a comprehensive and nationwide longitudinal investigation aimed at capturing the experiences of individuals aged 45 and older throughout China. This initiative commenced with a baseline assessment in 2011, employing a multistage stratified probability-proportional-to-size sampling approach to encompass over 17,000 participants drawn from around 10,000 households across 150 counties in 28 provinces. The survey is designed to gather a wide array of data pertaining to demographics, economic status, health conditions, pensions, and other pertinent domains via computer-assisted, in-person interviews that take place every 2 years. In addition, venous blood specimens were obtained in both 2011 and 2021 from fasting participants to measure glucose, TG, and cholesterol levels through accurate enzyme colorimetry methods. The extensive microdata set generated by CHARLS provides invaluable insights that are essential for interdisciplinary research focused on the phenomenon of population aging in China.

**Figure 1:**
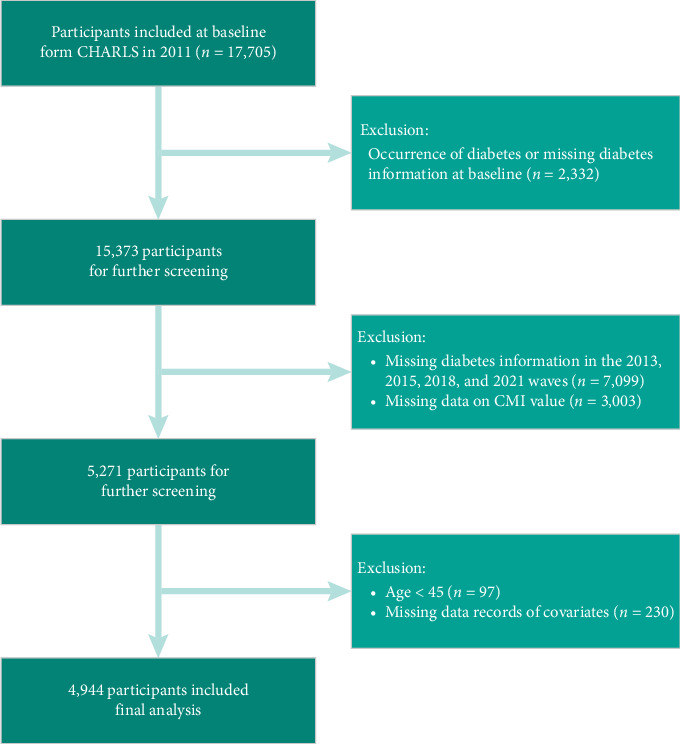
Flowchart for the selection of participants in the cohort study from CHARLS.

Figure 1 presents the flowchart detailing the selection process of participants. Initially, the study began with a cohort of 17,705 individuals who underwent physical examinations and completed questionnaire assessments during the baseline survey in 2011. However, several exclusions were necessary according to predefined criteria. Specifically, 2,332 individuals were excluded due to a prior diagnosis of diabetes at baseline, while 7,099 individuals were removed from the analysis because of missing diabetes data in the subsequent waves conducted in 2013, 2015, 2018, and 2020. Participants who died during the follow-up period were excluded from the analysis, in accordance with the CHARLS study protocol. Additionally, 3,003 individuals were omitted due to incomplete CMI data, 97 individuals were excluded for being younger than 45 years old, and 230 individuals were removed for lacking essential covariate information.  Table S2 demonstrates comparable baseline characteristics between excluded participants and the analytic cohort. Key metabolic indicators (CMI, TyG, atherogenic index of plasma [AIP]) showed minimal differences, with similar distributions in age, inflammatory markers (C-reactive protein [CRP], leucocytes), and comorbidity profiles (dyslipidemia, cardiovascular disease [CVD]). While some parameters reached statistical significance, the absolute differences were clinically negligible (< 5% relative difference for all retained variables).

### 2.2. Assessment of CMI

The subsequent equation was employed to calculate the CMI [6]:

(1) $$\begin{array}{c} \text{CMI}=\frac{\text{waist circumference }\left(\text{cm}\right)}{\text{height }\left(\text{cm}\right)}\times \frac{\text{TGs }\left(\text{mg}/\text{dL}\right)}{\text{HDL }\left(\text{mg}/\text{dL}\right)}. \end{array}$$

### 2.3. Assessment of New-Onset Diabetes Mellitus

In the present investigation, diabetes mellitus was classified based on specific criteria: a fasting blood glucose measurement of ≥ 126 mg/dL, a hemoglobin A1c (HbA1c) level of ≥ 6.5%, a self-reported diabetes diagnosis by a healthcare professional, or the administration of antidiabetic medication [19]. Participants who reported being diagnosed with diabetes mellitus by a healthcare professional were required to provide a fasting blood glucose measurement or HbA1c level to confirm the diagnosis. Those diagnosed during follow-up assessments were also asked to provide laboratory confirmation (fasting blood glucose or HbA1c level) for verification. Participants diagnosed with diabetes mellitus prior to 2011 were excluded from the analysis. Conversely, individuals diagnosed with diabetes mellitus during the follow-up assessments conducted in 2013, 2015, 2018, or 2021 were categorized as having newly developed diabetes mellitus in accordance with the definitions established for this study.

### 2.4. Assessment of Covariates

The evaluation of covariates entailed an exhaustive analysis of sociodemographic traits, lifestyle practices, and pre-existing health issues. Sociodemographic characteristics were categorized by age (in years), sex (male or female), educational attainment (ranging from middle school and above to primary/illiterate), and marital status (classified as married, divorced, widowed, or single). Lifestyle practices included smoking history (never smoked, former smoker, or current smoker) as well as alcohol consumption patterns. Current health conditions were classified as either present or absent, with a focus on hypertension, dyslipidemia, and CVD. Data from laboratory examinations encompassed blood urea nitrogen (BUN), creatinine levels, HbA1c, CRP, white blood cell count, and BMI. Dyslipidemia was defined by total cholesterol concentrations of 240 mg/dL or higher, TG levels of 150 mg/dL or more, low-density lipoprotein cholesterol (LDL-C) levels commencing at 160 mg/dL, and high-density lipoprotein cholesterol (HDL-C) levels under 40 mg/dL [20]. Hypertension was characterized by a systolic blood pressure (SBP) of at least 140 mmHg, diastolic blood pressure (DBP) of 90 mmHg or greater, ongoing utilization of antihypertensive therapies, or a self-reported diagnosis of hypertension [21]. Finally, CVD was evaluated based on a self-reported history of stroke or heart ailment [22]. Anthropometric measurements, including height, weight, and waist circumference, were taken by trained staff following standardized protocols. Inter-rater reliability was assessed through regular calibration of measurement instruments and consistency checks among raters, with high agreement observed in routine quality control assessments.

### 2.5. Statistical Analysis

The statistical analysis was carried out utilizing SPSS (Version 26.0) and R software (Version 4.2.1) to facilitate thorough data evaluation. Continuous variables are expressed as means accompanied by standard deviations (SDs) for data that follow a normal distribution, while medians with interquartile ranges (IQRs) are employed for data that do not adhere to normality. Categorical variables are represented through frequencies and percentages. To assess differences between groups, one-way ANOVA was utilized for continuous variables exhibiting a normal distribution, whereas the Kruskal–Wallis test was applied for non-normally distributed continuous data. The chi-square test was implemented to analyze disparities within categorical variables, supplemented by post hoc comparisons when deemed necessary. To investigate the relationship between baseline CMI levels and the emergence of new-onset diabetes mellitus, multivariate logistic regression models were employed. The initial analysis involved the computation of crude odds ratios (ORs) alongside 95% confidence intervals (CIs). Subsequently, adjusted models were formulated to account for potential confounding variables. Model 1 evaluated the association without any adjustments for additional covariates, establishing a baseline comparison. Model 2 incorporated a wider array of covariates, including age, gender, education, marital status, alcohol consumption, smoking habits, BMI, BUN, creatinine, HbA1c, CRP, and leucocyte count. Model 3 advanced the analysis by adding controls for hypertension, dyslipidemia, and CVD in addition to the variables already included in Model 2. To address potential nonlinearity in the association between CMI and diabetes risk, a restricted cubic spline analysis was applied. The predictive value of CMI for diabetes mellitus was assessed via the area under the receiver operating characteristic curve (AUC). Stratified analyses were performed across various subgroups (defined by sociodemographic factors and medical history) to ascertain the consistency of the relationship between CMI and diabetes. Furthermore, both univariable and multivariable logistic regression analyses identified independent predictors of new-onset diabetes. Mediation analysis examined the pathways through which CMI affects diabetes risk and quantified the proportion of the total effect mediated by each factor utilizing the product-of-coefficients approach. All statistical tests were conducted as two-sided, with a *p* value of less than 0.05 considered statistically significant.

## 3. Results

### 3.1. Study Participant Characteristics by New-Onset Diabetes Mellitus Status and CMI Quartiles

In this investigation, which involved a total of 4,944 individuals, 786 were identified as having diabetes during the follow-up assessment. Participants were followed for 2–9 years.  Table 1 highlights the baseline characteristics, showing that individuals with diabetes were generally older, with a significant proportion in the age groups over 60 years old, and had higher BMI values, particularly in the > 28 category. These participants also exhibited distinctive CMI distribution, predominantly falling in the highest quartile (Q4), along with elevated median levels of metabolic and inflammatory markers such as TyG, AIP, HbA1c, CRP, and leucocytes. A higher prevalence of hypertension, dyslipidemia, and CVD was observed among the diabetic group, suggesting unique metabolic and inflammatory profiles compared to nondiabetic individuals.  Table 2 further categorizes baseline characteristics by CMI quartiles, suggesting that elevated quartiles of CMI were more frequently observed in female participants and individuals from older age demographics, while also correlating with increased BMI. Elevated levels of TyG, AIP, CRP, leukocytes, and creatinine were consistently observed in higher CMI quartiles, which also had increased occurrences of hypertension, diabetes, dyslipidemia, and CVD, indicating a robust correlation between elevated CMI and heightened risk factors for these health conditions. A comparison of baseline characteristics between participants included in the analysis and those excluded can be seen in Supporting Table S1.

### 3.2. Relationship Between CMI and the Development of New-Onset Diabetes Mellitus

**Figure 2:**
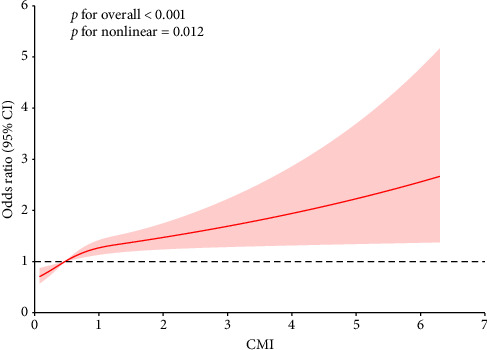
Adjusted cubic spline models examining the relationship between CMI and new-onset diabetes mellitus.

Table 3 illustrates the prospective relationships between the baseline CMI and the follow-up incidence of diabetes among participants in the CHARLS study, employing multivariate regression models for analysis. When contrasted with the reference group (Q1), a notable rise in diabetes incidence was observed with ascending CMI quartiles. In Model 1, the OR for Q4 was calculated at 2.41 (95% CI, 1.94 to 3.02; *p* < 0.001), signifying a 141% elevation in diabetes risk. This association remained consistently significant even after controlling for additional covariates in Models 2 and 3, yielding ORs of 1.77 (95% CI, 1.38 to 2.28; *p* < 0.001) and 1.73 (95% CI, 1.33 to 2.26; *p* < 0.001), respectively. The trend across the quartiles was statistically significant (*p* for trend < 0.001) in all models, highlighting the strong link between increased CMI and the heightened risk of diabetes development. Additionally, CMI was analyzed as a continuous variable, providing a more granular perspective on its association with diabetes risk. In Model 1, the continuous CMI variable was significantly associated with increased diabetes risk (OR = 1.37, 95% CI, 1.25 to 1.51; *p* < 0.001). The results remained significant in Model 2 (OR = 1.24, 95% CI, 1.13 to 1.36; *p* < 0.001) and Model 3 (OR = 1.24, 95% CI, 1.11 to 1.38; *p* < 0.001), further reinforcing the positive relationship between CMI and the risk of developing new-onset diabetes. Complementing these findings, Figure 2 depicts the relationship between CMI and diabetes incidence using fully adjusted cubic spline models. The analysis pinpoints a significant overall association (*p* < 0.001) and a nonlinear relationship (*p* = 0.012) between CMI levels and diabetes risk. This indicates that diabetes risk fluctuates across different CMI thresholds, underscoring the intricate metabolic influences on diabetes development within the fully adjusted model. This suggests that the CMI–diabetes linkage is more complex than a mere linear correlation, indicating specific CMI thresholds at which risk levels might disproportionately rise or fall, thereby underscoring the intricate role of CMI as a metabolic risk factor. The ROC curve analysis further supports the potential of CMI as a predictive biomarker for diabetes mellitus. The AUC value (0.596) suggests that CMI has a moderate discriminatory power for identifying individuals at risk of developing diabetes (Figure S1).

### 3.3. Stratified Analysis

**Figure 3:**
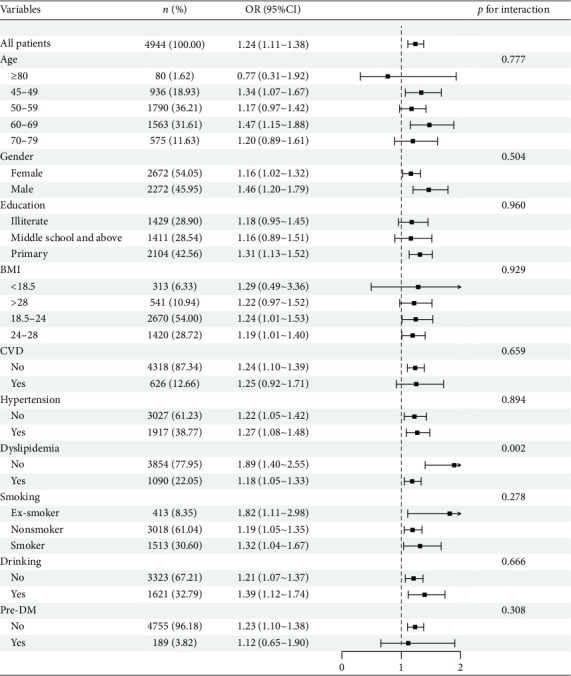
Forest plot of stratified analysis for the association between CMI and diabetes mellitus risk.

To assess the reliability of the positive correlation between the CMI and the incidence of new-onset diabetes mellitus, participants were categorized into subgroups according to their sociodemographic factors and medical history. The association was subsequently analyzed within each subgroup (see Figure 3). Additional interaction analyses indicated that dyslipidemia may serve as a moderating factor in the CMI's relationship with new-onset diabetes mellitus (*p* for interaction = 0.002). Specifically, for individuals diagnosed with dyslipidemia, each incremental rise in the CMI correlates with an 18% increase in the odds of developing diabetes mellitus, yielding an OR of 1.18 and a 95% CI ranging from 1.05 to 1.33. Conversely, among individuals without dyslipidemia, each unit increase in the CMI corresponds to an 89% elevation in the odds of diabetes mellitus, producing an OR of 1.89 and a 95% CI from 1.40 to 2.55.

### 3.4. Summary of Univariable and Multivariable Logistic Regression and Mediation Analysis Results

**Figure 4:**
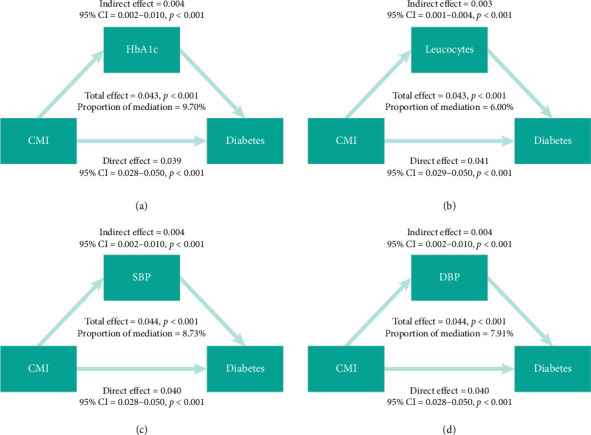
Results of mediation analysis depicting the pathways of key influential variables.

The findings from both univariate and multivariate logistic regression analyses are outlined in Supporting Table S2 and offer significant insights into the predictors of new-onset diabetes mellitus, particularly focusing on the role of the CMI. Univariate analysis identified significant factors, including age, BMI, CMI, HbA1c, leucocyte count, dyslipidemia, hypertension, and CVD. Multivariate analysis, adjusting for various confounders, confirmed age, BMI, CMI, HbA1c, and hypertension as significant predictors. These analyses lay the groundwork for the subsequent mediation analysis, results of which are illustrated in Figure 4. The mediation analysis examined intermediary factors such as HbA1c, leucocytes, SBP, and DBP. It was found that HbA1c mediated 9.70% of the association between CMI and the likelihood of developing new-onset diabetes mellitus. Additional mediation effects included leucocytes at 6.00%, SBP at 8.93%, and DBP at 7.91%. These findings inform targeted intervention strategies focusing on these mediators to reduce the incidence of diabetes.

## 4. Discussion

### 4.1. Comparison With Existing Research Results

The findings from our investigation correspond closely with an expanding collection of studies that identify CMI as a crucial predictor for the development of diabetes mellitus, reinforcing its clinical utility. Consistent with our findings, Ichiro et al. demonstrated the pivotal role of CMI in gauging hyperglycemia and diabetes risk, identifying significant associations between the highest quartiles of CMI with elevated HbA1c levels in both Japanese male and female populations [6]. ROC analysis in this study revealed strong discriminatory power, with ORs indicating a profound association of high CMI with diabetes, paralleling our observation that individuals in Q4 are at a markedly increased risk for diabetes compared to Q1. Shi et al.'s investigation also revealed that elevated CMI values are associated with a heightened risk of developing diabetes, regardless of gender. Intriguingly, the study's logistic regression analysis yielded ORs for diabetes development in the highest versus lowest CMI quartiles remarkably higher than ours (ORs of 3.736 for females and 3.697 for males compared to our OR of 2.41) [7].

Furthermore, our mediation analysis indicates that HbA1c and leukocyte count significantly mediate the connection between CMI and the risk of developing diabetes, a finding that resonates with Xu et al. who discovered substantial mediation impacts of inflammation markers in the link between CMI and diabetes risk [8]. Similarly, Liu et al.'s exploration confirmed multivariate regression indicating significant associations of CMI with glucose metabolic biomarkers, which our study supports with the observed mediating effects through biochemical markers such as HbA1c [23]. In the context of nonlinear associations, Qiu et al. also noted elevated diabetes risks with ascending CMI values in the CHARLS cohort, much like our study's findings [18]. A significant contribution from Qiu et al.'s research was highlighting risks associated with changes in CMI over time, suggesting that longitudinal CMI trajectories may bear substantial implications for diabetes risk evaluation. Further reinforcing the predictive power of CMI, Wu and Xu found significant correlations between CMI and IR, with ROC analyses placing CMI superior to traditional adiposity and lipid indices [24].

Lastly, Song et al. established an inverse L-shaped correlation between CMI and diabetes [16], suggesting the presence of a threshold effect that aligns with the observations made by Xu et al. [8] and Liu et al. [23]. Our study confirms the nature of this relationship and emphasizes age, BMI, and other comorbidities as significant factors interacting with CMI thresholds. The alignment of these results across various populations highlights the potential of CMI to serve as a crucial indicator for the stratification of diabetes risk, though variations in cutoff values and interaction effects highlight the need for further research to refine CMI's application in different epidemiological contexts. The stronger CMI–diabetes association in dyslipidemia-free individuals may reflect early metabolic deterioration: In these subjects, elevated CMI likely signifies emerging visceral adiposity and IR before overt dyslipidemia manifests. Conversely, in established dyslipidemia, additional factors (e.g., statin use, genetic hyperlipidemia) may dilute CMI's predictive specificity. Our study not only corroborates the extant literature but also expands on it, emphasizing the intriguing interplay of systemic inflammatory markers and hypertension as moderators and mediators—factors that can crucially influence CMI's predictive scope and, ultimately, diabetes management strategies.

The robust association between elevated CMI and diabetes risk may be mediated through interconnected pathophysiological pathways. CMI integrates two key components: (1) visceral adiposity (via waist-to-height ratio) and (2) atherogenic dyslipidemia (via TG/HDL ratio). Visceral adipose tissue drives IR through chronic inflammation (e.g., increased TNF-α, IL-6) and altered adipokine secretion (e.g., reduced adiponectin). Concurrently, dyslipidemia exacerbates lipotoxicity, promoting ectopic lipid deposition in the liver and skeletal muscle, which impairs insulin signaling and β-cell function. Our mediation analysis supports this framework, showing that HbA1c (reflecting chronic hyperglycemia) and inflammatory markers (leucocytes) partially mediate the CMI–diabetes relationship. These pathways collectively promote β-cell dysfunction and progressive hyperglycemia, ultimately culminating in diabetes.

### 4.2. Strengths and Limitations

This research is underpinned by several significant advantages. Primarily, the utilization of longitudinal data derived from the CHARLS cohort, encompassing a substantial and nationally representative sample of Chinese individuals aged 45 years and above, bolsters the external validity of our results. The inclusion of both baseline characteristics and the longitudinal follow-up of participants over several years allows us to make stronger inferences about the causal relationship between CMI and new-onset diabetes [25]. Moreover, the use of a comprehensive CMI that incorporates multiple risk factors provides a more holistic view of metabolic health than traditional single-marker models, thus improving the accuracy of risk prediction [26, 27]. Second, the robustness of our investigation is enhanced by the incorporation of multivariate logistic regression alongside stratified analyses, which effectively address potential confounding variables and modifiers of effect. This provides a more nuanced understanding of how CMI interacts with other health factors, contributing to the development of diabetes. Additionally, the mediation analysis offers valuable insight into the potential mechanisms through which CMI influences diabetes risk, furthering our understanding of the underlying pathophysiology.

Nonetheless, several limitations must be taken into account. To begin with, the observational design inherently restricts the ability to establish causality between CMI and new-onset diabetes mellitus, emphasizing the need for experimental validation through future studies. While our longitudinal design strengthens the causal inference between CMI and incident diabetes, we acknowledge that reverse causality remains a potential concern. Specifically, subclinical diabetes may influence CMI values, leading to the possibility that changes in CMI over time could reflect the early stages of undiagnosed diabetes. Future research incorporating more precise diagnostic tools for subclinical diabetes and longer follow-up periods may help further elucidate the direction of causality between CMI and diabetes. Despite the adjustments implemented for established confounding variables, including age, BMI, and hypertension, residual confounding continues to pose a significant concern. It is plausible that there exist unmeasured variables, such as genetic predispositions, family history of diabetes, physical activity levels, dietary patterns, medication use (e.g., lipid-lowering drugs or antihypertensives that may alter CMI components), or psychosocial factors, which may also play a role in influencing the risk of developing diabetes [28, 29]. Moreover, although HbA1c was controlled as an indicator of glucose metabolism, the lack of direct IR measures (such as HOMA-IR or hyperinsulinemic–euglycemic clamps) limits our understanding of mechanistic pathways. Incorporating more detailed measures of IR or pancreatic function could yield additional insights into the mechanisms linking CMI to diabetes. The cross-sectional nature of the baseline data further complicates the establishment of causality, despite the longitudinal design offering stronger causal inference. The potential for reverse causality, where the onset of diabetes may influence CMI scores over time, cannot be entirely dismissed. Although our follow-up period (2–9 years) aligns with peak diabetes incidence in aging populations, it may not capture very long-term progression beyond 9 years. Post hoc power analyses confirmed adequate power (> 99%) for primary analyses and major subgroups (e.g., nondyslipidemia). However, smaller strata (e.g., dyslipidemia subgroup) had reduced power (78%), potentially limiting detection of subtle associations. Ultimately, the demographic characteristics of the sample, which predominantly includes middle-aged and elderly individuals from China, constrain the applicability of the results to wider populations. Furthermore, although the CHARLS dataset includes genetic data, this analysis did not incorporate genetic factors due to the complexity of genetic analyses and the scope of the current study. However, we acknowledge that exploring gene–environment interactions between genetic predisposition and CMI could provide important mechanistic insights into the development of diabetes. Future studies utilizing the available genetic data could further strengthen the understanding of how genetic factors interact with metabolic indicators like CMI to influence diabetes risk.

### 4.3. Implications for Practice and Research

The results of this investigation hold significant relevance for clinical applications as well as subsequent research endeavors. Our findings suggest that CMI could be a valuable screening tool for identifying individuals at increased risk of diabetes. Based on the association observed in this study, individuals in the highest quartile (Q4) of CMI appear to be at significantly higher risk, which could warrant closer monitoring and early intervention. We recommend further investigation into specific CMI thresholds for diabetes risk stratification, particularly for adults aged 45 and older. However, we acknowledge that these thresholds may vary across different populations, and additional validation studies are needed to refine these cutoffs. Healthcare professionals should consider integrating CMI into routine screenings, especially for individuals who fall into higher-risk categories based on traditional risk factors, such as age, BMI, and family history of diabetes. Clinically, the CMI serves as an accessible and economical screening instrument designed to detect adults aged 45 and older who are at an increased risk of developing diabetes. Integrating CMI into standard health evaluations could enable healthcare professionals to identify individuals at risk more promptly, facilitating timely interventions that aim to prevent or postpone the development of diabetes [11]. For healthcare systems, this approach may help reduce the burden of diabetes by targeting high-risk populations for lifestyle interventions, such as physical activity promotion, dietary changes, and weight management [30]. Additionally, it could support the implementation of personalized prevention programs, as individuals with elevated CMI scores may require more intensive monitoring and preventive measures. In terms of research, future studies should focus on validating the CMI as a predictive tool in different populations to assess its generalizability. Moreover, additional studies are required to investigate the fundamental biological mechanisms that associate heightened CMI with a greater susceptibility to diabetes. This exploration should encompass genetic, hormonal, and inflammatory pathways [31]. Longitudinal studies that track changes in CMI over time and its relationship with other outcomes, such as CVD, would also be valuable in establishing the broader utility of the CMI as a marker of overall cardiometabolic health [5]. Finally, more work is needed to refine CMIs and assess their predictive accuracy in comparison with other risk factors, such as genetic markers or emerging biomarkers like gut microbiota profiles, which may also contribute to diabetes risk [32, 33]. This could lead to the development of multidimensional risk assessment tools that combine both traditional and novel risk markers for more precise prediction and prevention strategies. We acknowledge that machine learning approaches, such as random forests or support vector machines, could offer valuable insights into the predictive capacity of CMI, particularly in comparison with traditional risk factors. While our current study relied on conventional statistical methods, we plan to explore the use of machine learning models in future studies to validate our findings and assess whether CMI provides additional predictive value. This approach may further refine risk prediction and guide more personalized prevention strategies.

## 5. Conclusion

While the CMI demonstrates potential as a tool for assessing patient risk, its clinical utility requires careful consideration. In comparison with existing risk scores, the CMI provides an alternative approach that incorporates additional clinical factors which may offer a more tailored risk assessment for certain patient populations. However, the advantages of CMI over these established risk scores are not yet fully established, and further validation in diverse clinical settings is required. For practitioners, we recommend using CMI as a supplementary tool rather than a replacement for existing risk scores. It can be valuable for identifying at-risk patients, especially in contexts where current risk scores may be less effective. Nonetheless, its use should be guided by clinical judgment and the specific needs of the patient population. Future studies should focus on refining CMI and establishing clear, actionable guidelines for its integration into routine clinical practice.

## Figures and Tables

**Table 1:** Baseline characteristics of the study population grouped by diabetes status at follow-up.

	Total (*n* = 4944)	Diabetes (*n* = 786)	Nondiabetes (*n* = 4158)	*p*
Age (*n* (%))				< 0.001
45–49	80 (1.6)	14 (1.8)	66 (1.6)	
50–59	936 (18.9)	104 (13.2)	832 (20.0)	
60–69	1790 (36.2)	291 (37.0)	1499 (36.1)	
70–79	1563 (31.6)	265 (33.7)	1298 (31.2)	
≥ 80	575 (11.6)	112 (14.2)	463 (11.1)	
Gender (*n* (%))				0.601
Female	2672 (54.0)	432 (55.0)	2240 (53.9)	
Male	2272 (46.0)	354 (45.0)	1918 (46.1)	
Education (*n* (%))				0.163
Middle school and above	1411 (28.5)	206 (26.2)	1205 (29.0)	
Primary	2104 (42.6)	334 (42.5)	1770 (42.6)	
Illiterate	1429 (28.9)	246 (31.3)	1183 (28.5)	
Marital (*n* (%))				0.037
Married	4408 (89.2)	683 (86.9)	3725 (89.6)	
Divorced	39 (0.8)	5 (0.6)	34 (0.8)	
Widowed	467 (9.4)	95 (12.1)	372 (8.9)	
Unmarried	30 (0.6)	3 (0.4)	27 (0.6)	
BMI (*n* (%))				< 0.001
< 18.5	313 (6.3)	45 (5.7)	268 (6.4)	
18.5–24	541 (10.9)	148 (18.8)	393 (9.5)	
24–28	2670 (54.0)	332 (42.2)	2338 (56.2)	
> 28	1420 (28.7)	261 (33.2)	1159 (27.9)	
Smoking (%)				0.991
Nonsmoker	3018 (61.0)	481 (61.2)	2537 (61.0)	
Ex-smoker	413 (8.4)	66 (8.4)	347 (8.3)	
Smoker	1513 (30.6)	239 (30.4)	1274 (30.6)	
Drinking (%)				0.208
Yes	1621 (32.8)	242 (30.8)	1379 (33.2)	
No	3323 (67.2)	544 (69.2)	2779 (66.8)	
CMI (%)				< 0.001
Q1	1236 (25.0)	136 (17.3)	1100 (26.5)	
Q2	1236 (25.0)	167 (21.2)	1069 (25.7)	
Q3	1236 (25.0)	199 (25.3)	1037 (24.9)	
Q4	1236 (25.0)	284 (36.1)	952 (22.9)	
TyG (median (IQR))	4.0 (3.7, 4.4)	4.2 (3.9, 4.6)	4.0 (3.7, 4.4)	< 0.001
AIP (median (IQR))	−0.1 (−0.6, 0.4)	0.0 (−0.4, 0.6)	−0.2 (−0.6, 0.3)	< 0.001
BUN (median (IQR))	15.1 (12.5, 18.1)	14.7 (12.4, 17.7)	15.1 (12.5, 18.1)	0.192
Creatinine (median (IQR))	0.7 (0.6, 0.9)	0.8 (0.7, 0.9)	0.7 (0.6, 0.9)	0.255
HbA1c (median (IQR))	5.1 (4.9, 5.4)	5.3 (5.0, 5.5)	5.1 (4.8, 5.3)	< 0.001
CRP (median (IQR))	1.0 (0.5, 2.0)	1.2 (0.6, 2.5)	0.9 (0.5, 1.9)	< 0.001
Leucocytes (median (IQR))	5.9 (4.9, 7.2)	6.2 (5.1, 7.6)	5.9 (4.9, 7.1)	< 0.001
Hypertension (%)				< 0.001
Yes	1917 (38.8)	395 (50.3)	1522 (36.6)	
No	3027 (61.2)	391 (49.7)	2636 (63.4)	
Dyslipidemia (%)				< 0.001
Yes	1090 (22.0)	215 (27.4)	875 (21.0)	
No	3854 (78.0)	571 (72.6)	3283 (79.0)	
CVD (%)				0.001
Yes	626 (12.7)	128 (16.3)	498 (12.0)	
No	4318 (87.3)	658 (83.7)	3660 (88.0)	

**Table 2:** Baseline characteristics of the study population categorized by CMI quartiles.

	Total (*n* = 4944)	Q1 (*n* = 1236)	Q2 (*n* = 1236)	Q3 (*n* = 1236)	Q4 (*n* = 1236)	*p*
Age (*n* (%))						0.01
45–49	80 (1.6)	19 (1.5)	25 (2.0)	21 (1.7)	15 (1.2)	
50–59	936 (18.9)	231 (18.7)	234 (18.9)	225 (18.2)	246 (19.9)	
60–69	1790 (36.2)	414 (33.5)	446 (36.1)	448 (36.2)	482 (39.0)	
70–79	1563 (31.6)	394 (31.9)	381 (30.8)	406 (32.8)	382 (30.9)	
≥ 80	575 (11.6)	178 (14.4)	150 (12.1)	136 (11.0)	111 (9.0)	
Gender (*n* (%))						< 0.001
Female	2672 (54.0)	561 (45.4)	677 (54.8)	701 (56.7)	733 (59.3)	
Male	2272 (46.0)	675 (54.6)	559 (45.2)	535 (43.3)	503 (40.7)	
Education (*n* (%))						0.772
Middle school and above	1411 (28.5)	341 (27.6)	343 (27.8)	363 (29.4)	364 (29.4)	
Primary	2104 (42.6)	539 (43.6)	534 (43.2)	505 (40.9)	526 (42.6)	
Illiterate	1429 (28.9)	356 (28.8)	359 (29.0)	368 (29.8)	346 (28.0)	
Marital (*n* (%))						0.38
Married	4408 (89.2)	1095 (88.6)	1103 (89.2)	1097 (88.8)	1113 (90.0)	
Divorced	39 (0.8)	12 (1.0)	7 (0.6)	8 (0.6)	12 (1.0)	
Widowed	467 (9.4)	116 (9.4)	121 (9.8)	124 (10.0)	106 (8.6)	
Unmarried	30 (0.6)	13 (1.1)	5 (0.4)	7 (0.6)	5 (0.4)	
BMI (*n* (%))						< 0.001
< 18.5	313 (6.3)	163 (13.2)	95 (7.7)	35 (2.8)	20 (1.6)	
18.5–24	541 (10.9)	25 (2.0)	66 (5.3)	160 (12.9)	290 (23.5)	
24–28	2670 (54.0)	882 (71.4)	751 (60.8)	620 (50.2)	417 (33.7)	
> 28	1420 (28.7)	166 (13.4)	324 (26.2)	421 (34.1)	509 (41.2)	
Smoking (*n* (%))						< 0.001
Nonsmoker	3018 (61.0)	678 (54.9)	755 (61.1)	784 (63.4)	801 (64.8)	
Ex-smoker	413 (8.4)	110 (8.9)	98 (7.9)	100 (8.1)	105 (8.5)	
Smoker	1513 (30.6)	448 (36.2)	383 (31.0)	352 (28.5)	330 (26.7)	
Drinking (*n* (%))						< 0.001
Yes	1621 (32.8)	508 (41.1)	401 (32.4)	365 (29.5)	347 (28.1)	
No	3323 (67.2)	728 (58.9)	835 (67.6)	871 (70.5)	889 (71.9)	
TyG (median (IQR))	4.0 (3.7, 4.4)	3.5 (3.3, 3.7)	3.9 (3.7, 4.0)	4.2 (4.0, 4.4)	4.7 (4.5, 5.0)	< 0.001
AIP (median (IQR))	−0.1 (−0.6, 0.4)	−0.9 (−1.1, −0.7)	−0.3 (−0.5, −0.2)	0.1 (0.0, 0.2)	0.8 (0.5, 1.1)	< 0.001
BUN (median (IQR))	15.1 (12.5, 18.1)	16.0 (13.1, 19.2)	15.0 (12.7, 18.2)	14.7 (12.5, 17.6)	14.5 (12.2, 17.2)	< 0.001
Creatinine (median (IQR))	0.7 (0.6, 0.9)	0.7 (0.6, 0.9)	0.7 (0.6, 0.9)	0.7 (0.6, 0.9)	0.8 (0.7, 0.9)	0.005
HbA1c (median (IQR))	5.1 (4.9, 5.4)	5.1 (4.8, 5.3)	5.1 (4.9, 5.3)	5.1 (4.8, 5.4)	5.1 (4.9, 5.4)	< 0.001
CRP (median (IQR))	1.0 (0.5, 2.0)	0.8 (0.4, 1.6)	0.8 (0.5, 1.8)	1.0 (0.6, 2.0)	1.3 (0.7, 2.5)	< 0.001
Leucocytes (median (IQR))	5.9 (4.9, 7.2)	5.6 (4.7, 6.7)	5.8 (4.8, 7.1)	6.0 (4.9, 7.2)	6.3 (5.3, 7.6)	< 0.001
Hypertension (*n* (%))						< 0.001
Yes	1917 (38.8)	362 (29.3)	410 (33.2)	526 (42.6)	619 (50.1)	
No	3027 (61.2)	874 (70.7)	826 (66.8)	710 (57.4)	617 (49.9)	
Diabetes (*n* (%))						< 0.001
Yes	786 (15.9)	136 (11.0)	167 (13.5)	199 (16.1)	284 (23.0)	
No	4158 (84.1)	1100 (89.0)	1069 (86.5)	1037 (83.9)	952 (77.0)	
Dyslipidemia (*n* (%))						< 0.001
Yes	1090 (22.0)	109 (8.8)	149 (12.1)	190 (15.4)	642 (51.9)	
No	3854 (78.0)	1127 (91.2)	1087 (87.9)	1046 (84.6)	594 (48.1)	
CVD (*n* (%))						< 0.001
Yes	626 (12.7)	133 (10.8)	137 (11.1)	161 (13.0)	195 (15.8)	
No	4318 (87.3)	1103 (89.2)	1099 (88.9)	1075 (87.0)	1041 (84.2)	

**Table 3:** Prospective association between baseline CMI and incident diabetes during follow-up in CHARLS.

	Model 1	*p*	Model 2	*p*	Model 3	*p*
CMI	1.37 (1.25, 1.51)	< 0.001	1.24 (1.13, 1.36)	< 0.001	1.24 (1.11, 1.38)	< 0.001
Q1	Ref		Ref		Ref	
Q2	1.26 (0.99, 1.61)	0.058	1.13 (0.88, 1.45)	0.328	1.13 (0.88, 1.46)	0.320
Q3	1.55 (1.23, 1.96)	< 0.001	1.29 (1.01, 1.66)	0.043	1.27 (0.99, 1.63)	0.061
Q4	2.41 (1.94, 3.02)	< 0.001	1.77 (1.38, 2.28)	< 0.001	1.73 (1.33, 2.26)	< 0.001
*p* for trend	< 0.001		< 0.001		< 0.001	

## Data Availability

The data underpinning the conclusions of this research can be accessed through the China Health and Retirement Longitudinal Study website (https://charls.pku.edu.cn/). However, access to these data is subject to certain restrictions, as they were utilized under a specific license for this study and are consequently not publicly accessible. Nevertheless, data can be obtained from the corresponding author upon a reasonable request and with the approval of the CHARLS research team.

## Supporting Information

Table S1: Baseline characteristics comparison between participants excluded due to missing diabetes data and the analytic cohort.

Table S2: Results of univariable and multivariable logistic regression for new-onset diabetes.

Figure S1: Receiver operating characteristic (ROC) curve for cardiometabolic index (CMI) in predicting new-onset diabetes.
