# Supplementary material for: The Relationship Between Cardiometabolic Index and New-Onset Diabetes in Adults Aged Over 45: A Longitudinal Analysis Based on CHARLS
Source: Int J Endocrinol. 2025 Jul 29;2025:2259853. doi: 10.1155/ije/2259853 (PMC12324913; doi:10.1155/ije/2259853)
Supplement: Supporting Information — Additional supporting information can be found online in the Supporting Information section. [file 2259853.f1.docx]

Supplementary Material

## Table S1 Baseline characteristics comparison between participants excluded due to missing diabetes data and the analytic cohort.

| **Variables** | **Total (n = 12043)** | **Excluded (n = 7099)** | **Included (n = 4944)** | **P** |
| --- | --- | --- | --- | --- |
| **Age, M (Q₁, Q₃)** | 58.00 (51.00, 66.00) | 58.00 (50.00, 67.00) | 58.00 (52.00, 65.00) | 0.621 |
| **Gender, n(%)** |  |  |  | <.001 |
| Female | 6243 (51.85) | 3571 (50.32) | 2672 (54.05) |  |
| Male | 5797 (48.15) | 3525 (49.68) | 2272 (45.95) |  |
| **Education, n(%)** |  |  |  | <.001 |
| Illiterate | 3321 (27.65) | 1892 (26.77) | 1429 (28.90) |  |
| Middle school and above | 3999 (33.29) | 2588 (36.62) | 1411 (28.54) |  |
| primary | 4692 (39.06) | 2588 (36.62) | 2104 (42.56) |  |
| **Marital, n(%)** |  |  |  | <.001 |
| divorced | 172 (1.43) | 133 (1.88) | 39 (0.79) |  |
| married | 10415 (86.63) | 6007 (84.86) | 4408 (89.16) |  |
| unmarried | 111 (0.92) | 81 (1.14) | 30 (0.61) |  |
| widowed | 1325 (11.02) | 858 (12.12) | 467 (9.45) |  |
| **BMI, M (Q₁, Q₃)** | 22.90 (20.64, 25.43) | 22.72 (20.43, 25.22) | 23.07 (20.85, 25.65) | <.001 |
| **Smoking, n(%)** |  |  |  | 0.005 |
| Ex-smoker | 959 (8.32) | 546 (8.30) | 413 (8.35) |  |
| Non-smoker | 7218 (62.62) | 4200 (63.81) | 3018 (61.04) |  |
| Smoker | 3349 (29.06) | 1836 (27.89) | 1513 (30.60) |  |
| **Drinking, n(%)** |  |  |  | 0.460 |
| No | 7976 (66.83) | 4653 (66.57) | 3323 (67.21) |  |
| Yes | 3958 (33.17) | 2337 (33.43) | 1621 (32.79) |  |
| **BUN, M (Q₁, Q₃)** | 15.10 (12.52, 18.21) | 15.15 (12.49, 18.35) | 15.07 (12.55, 18.07) | 0.410 |
| **Creatinine, M (Q₁, Q₃)** | 0.76 (0.66, 0.88) | 0.77 (0.66, 0.90) | 0.75 (0.64, 0.87) | <.001 |
| **CRP, M (Q₁, Q₃)** | 0.98 (0.53, 2.05) | 1.00 (0.53, 2.14) | 0.96 (0.53, 1.99) | 0.052 |
| **CMI, M (Q₁, Q₃)** | 0.47 (0.28, 0.80) | 0.45 (0.27, 0.78) | 0.47 (0.29, 0.81) | 0.010 |
| **HbA1c, M (Q₁, Q₃)** | 5.10 (4.80, 5.30) | 5.10 (4.80, 5.30) | 5.10 (4.90, 5.40) | 0.002 |
| **Leucocytes, M (Q₁, Q₃)** | 5.90 (4.90, 7.20) | 5.93 (4.90, 7.20) | 5.90 (4.90, 7.20) | 0.570 |
| **TyG, M (Q₁, Q₃)** | 4.04 (3.69, 4.43) | 4.03 (3.69, 4.42) | 4.04 (3.70, 4.43) | 0.285 |
| **AIP, M (Q₁, Q₃)** | -0.13 (-0.58, 0.38) | -0.13 (-0.59, 0.38) | -0.13 (-0.58, 0.37) | 0.971 |
| **Hypertension, n(%)** |  |  |  | <.001 |
| No | 5939 (57.98) | 2912 (54.94) | 3027 (61.23) |  |
| Yes | 4305 (42.02) | 2388 (45.06) | 1917 (38.77) |  |
| **Dyslipidemia, n(%)** |  |  |  | 0.288 |
| No | 6661 (78.36) | 2807 (78.91) | 3854 (77.95) |  |
| Yes | 1840 (21.64) | 750 (21.09) | 1090 (22.05) |  |
| **CVD, n(%)** |  |  |  | 0.766 |
| No | 10383 (87.23) | 6065 (87.15) | 4318 (87.34) |  |
| Yes | 1520 (12.77) | 894 (12.85) | 626 (12.66) |  |

## Table S2 Univariable and multivariable logistic regression.

| **Variables** | **univariable logistic regression** | | | | |  | **multivariable logistic regression** | | | | |
| --- | --- | --- | --- | --- | --- | --- | --- | --- | --- | --- | --- |
|  | **β** | **SE** | **Z** | ***P*** | **OR (95%CI)** |  | **β** | **SE** | **Z** | ***P*** | **OR (95%CI)** |
| **Age** | 0.01 | 0.01 | 2.63 | 0.009 | 1.01 (1.01 ~ 1.02) |  | 0.01 | 0.01 | 2.22 | 0.026 | 1.01 (1.01 ~ 1.02) |
| **Gender** |  |  |  |  |  |  |  |  |  |  |  |
| **Female** |  |  |  |  | Reference |  |  |  |  |  |  |
| **Male** | -0.11 | 0.10 | -1.11 | 0.266 | 0.90 (0.75 ~ 1.08) |  |  |  |  |  |  |
| **Education** |  |  |  |  |  |  |  |  |  |  |  |
| **illiterate** |  |  |  |  | Reference |  |  |  |  |  |  |
| **Middle school and above** | -0.12 | 0.12 | -0.95 | 0.343 | 0.89 (0.70 ~ 1.13) |  |  |  |  |  |  |
| **primary** | -0.06 | 0.11 | -0.57 | 0.571 | 0.94 (0.75 ~ 1.17) |  |  |  |  |  |  |
| **Marital** |  |  |  |  |  |  |  |  |  |  |  |
| **divorced** |  |  |  |  | Reference |  |  |  |  |  |  |
| **married** | 0.74 | 0.74 | 1.00 | 0.317 | 2.09 (0.49 ~ 8.87) |  |  |  |  |  |  |
| **unmarried** | 0.23 | 1.05 | 0.22 | 0.823 | 1.26 (0.16 ~ 9.81) |  |  |  |  |  |  |
| **widowed** | 1.06 | 0.75 | 1.42 | 0.156 | 2.89 (0.67 ~ 12.56) |  |  |  |  |  |  |
| **BMI** | 0.09 | 0.01 | 7.26 | <.001 | 1.09 (1.07 ~ 1.12) |  | 0.06 | 0.01 | 4.44 | <.001 | 1.06 (1.03 ~ 1.09) |
| **Smoking** |  |  |  |  |  |  |  |  |  |  |  |
| **Ex-smoker** |  |  |  |  | Reference |  |  |  |  |  |  |
| **Non-smoker** | 0.16 | 0.18 | 0.91 | 0.362 | 1.18 (0.83 ~ 1.67) |  |  |  |  |  |  |
| **Smoker** | 0.09 | 0.19 | 0.46 | 0.647 | 1.09 (0.75 ~ 1.58) |  |  |  |  |  |  |
| **Drinking** |  |  |  |  |  |  |  |  |  |  |  |
| **No** |  |  |  |  | Reference |  |  |  |  |  |  |
| **Yes** | -0.18 | 0.10 | -1.72 | 0.085 | 0.84 (0.69 ~ 1.02) |  |  |  |  |  |  |
| **BUN** | -0.01 | 0.01 | -0.96 | 0.339 | 0.99 (0.97 ~ 1.01) |  |  |  |  |  |  |
| **Creatinine** | 0.05 | 0.26 | 0.20 | 0.840 | 1.05 (0.63 ~ 1.76) |  |  |  |  |  |  |
| **CRP** | 0.01 | 0.01 | 1.05 | 0.294 | 1.01 (1.00 ~ 1.02) |  |  |  |  |  |  |
| **CMI** | 0.33 | 0.06 | 5.83 | <.001 | 1.39 (1.25 ~ 1.56) |  | 0.21 | 0.06 | 3.62 | <.001 | 1.24 (1.10 ~ 1.39) |
| **HbA1c** | 1.27 | 0.13 | 10.17 | <.001 | 3.57 (2.80 ~ 4.57) |  | 1.16 | 0.13 | 9.06 | <.001 | 3.20 (2.49 ~ 4.12) |
| **Leucocytes** | 0.09 | 0.02 | 3.77 | <.001 | 1.09 (1.04 ~ 1.14) |  | 0.05 | 0.03 | 1.90 | 0.057 | 1.05 (1.00 ~ 1.10) |
| **Dyslipidemia** |  |  |  |  |  |  |  |  |  |  |  |
| **No** |  |  |  |  | Reference |  |  |  |  |  |  |
| **Yes** | 0.37 | 0.11 | 3.48 | <.001 | 1.45 (1.18 ~ 1.79) |  |  |  |  |  |  |
| **Hypertension** |  |  |  |  |  |  |  |  |  |  |  |
| **No** |  |  |  |  | Reference |  |  |  |  |  | Reference |
| **Yes** | 0.60 | 0.10 | 6.25 | <.001 | 1.81 (1.50 ~ 2.19) |  | 0.39 | 0.10 | 3.78 | <.001 | 1.48 (1.21 ~ 1.81) |
| **CVD** |  |  |  |  |  |  |  |  |  |  |  |
| **No** |  |  |  |  | Reference |  |  |  |  |  |  |
| **Yes** | 0.38 | 0.13 | 2.93 | 0.003 | 1.46 (1.13 ~ 1.88) |  |  |  |  |  |  |


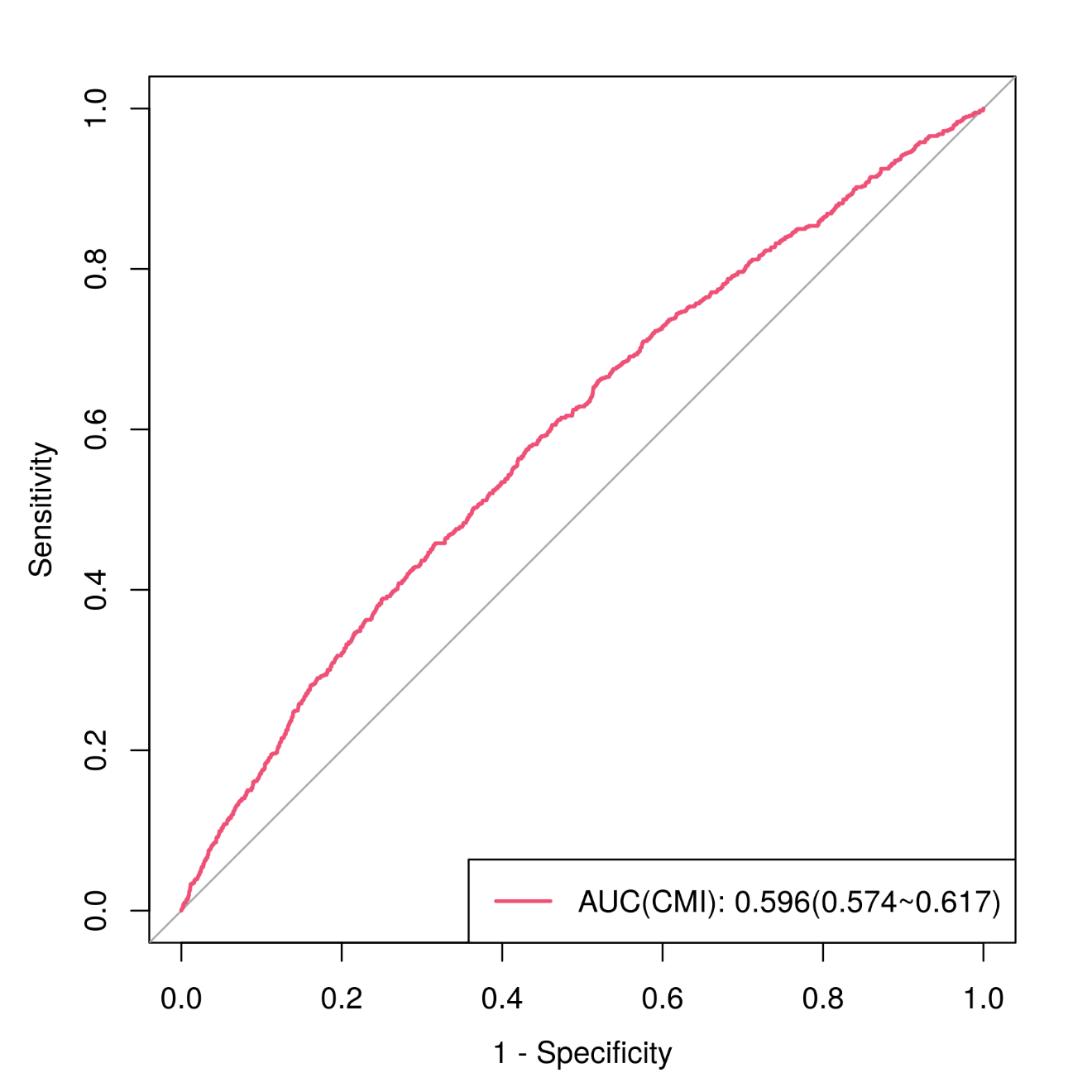


**Fig. S1 The receiver operating characteristic curve.**
